# An adaptive gene-based test for methylation data

**DOI:** 10.1186/s12919-018-0126-9

**Published:** 2018-09-17

**Authors:** Chong Wu, Jun Young Park, Weihua Guan, Wei Pan

**Affiliations:** Division of Biostatistics, University of Minnesota, 420 Delaware St SE, Minneapolis, MN 55455 USA

## Abstract

DNA methylation plays an important role in normal human development and disease. In epigenome-wide association studies (EWAS), a univariate test for association between a phenotype and each cytosine-phosphate-guanine (CpG) site has been widely used. Given the number of CpG sites tested in EWAS, a stringent significance cutoff is required to adjust for multiple testing; in addition, multiple nearby CpG sites may be associated with the phenotype, which is ignored by a univariate test. These two factors may contribute to the power loss of a univariate test. As an alternative, we propose applying an adaptive gene-based test that is powerful in genome-wide association studies (GWAS), called *aSPUw*, to EWAS for simultaneous testing on multiple CpG sites within or near a gene. We show its application to the GAW20 methylation data set.

## Background

DNA methylation of cytosine residues at cytosine-phosphate-guanine (CpG) dinucleotides is of particular interest because it has a central role in the normal human development and disease [1]. Epigenome-wide association studies (EWAS), analogous to genome-wide association studies (GWAS), are becoming increasingly popular to interrogate methylation changes associated with a disease or related environmental factors [2]. The common statistical analysis in EWAS uses a single marker test for association between a phenotype and each of the CpG sites. Given the number of CpG sites tested in EWAS, a univariate test must meet a stringent threshold for statistical significance (for example, *p* value < 1 × 10^− 7^ often used for the Illumina 450 K array); in addition, a univariate test does not take advantage of possible existence of multiple associated CpG sites within a gene. Hence, a univariate test many be underpowered in EWAS. In this paper, we consider statistical methods that test for the association between multiple CpG sites in a gene and a phenotype simultaneously.

This work was motivated by analysis of the methylation data provided by GAW20. The data set was taken from the Genetics of Lipid Lowering Drugs and Diet Network (GOLDN) study. With GOLDN data, Irvin et al. found 4 CpG sites in intron 1 of *CPT1A* that were strongly associated with both very-low-density lipoprotein cholesterol (VLDL-C) and triglyceride (TG) [3]. A question of major interest is to study whether other genes are associated with VLDL-C and TG. In other words, we are interested in examining the association between methylation within each gene and a phenotype.

Some methods have been developed to detect differentially methylated regions (DMRs) [4, 5]. For example, Jaffe et al. [4] proposed a method called *bump hunting* to detect DMRs associated with a continuous trait in a well-characterized population of newborns. Butcher and Beck [5] proposed a flexible window-based approach to discover DMRs. Many methods detecting DMRs assume that nearby CpG sites are methylated/unmethylated in the same direction, however, to the best of our knowledge, few gene-based analysis for methylation data consider the scenario that the change of methylation status can be in different directions within a gene. Importantly, because it is reasonable to assume that not all the CpG sites in a gene are related to the phenotype, it is challenging and important to adaptively aggregate information over multiple associated CpG sites while eliminating or minimizing the effects of nonassociated CpG sites. To solve this problem, we apply a highly adaptive test called *adaptive sum of powered score (aSPU)* test [6] and its weighted version called *aSPUw* test [7], which have promising performance in GWAS, to methylation data for the first time. The main idea of the aSPU or aSPUw test is that, because we do not know which and how many CpG sites in the given gene are associated with the phenotype, we construct a class of sum of powered score–weighted (SPUw) tests. The aSPUw test then selects the CpG site with the most significant result with a proper adjustment for multiple testing.

In application to the GAW20 data set, to account for familial structures, we apply a linear mixed-effects model and use an estimated genetic relationship matrix (GRM) (estimating the kinship coefficients among the individuals) as the correlation matrix of a random effect. Then we construct the aSPUw test based on the score function from the linear mixed-effects model. We find that methylation levels in gene *CPT1A* are associated with the pretreatment fasting TGs at the genome-wide significance level, whereas methylation levels in gene *APOA5* are possibly associated at a suggestive significance level.

## Methods

In this section, we briefly introduce the aSPUw test for family-based methylation data.

Suppose for *n*_*i*_ related family members in the family *i* (*i* = 1, 2, …, *F*), we have an *n*_*i*_-vector ${y}_i={\left({y}_{i1},\dots, {y}_{in_i}\right)}^{\prime }$ for a phenotype, for example, log pretreatment fasting TG levels, an *n*_*i*_ × *p* matrix *G*_*i*_ = (*G*_*i*1_, …, *G*_*ip*_)^′^ for for *p* CpG sites, and an *n*_*i*_ × *q* matrix *X*_*i*_ = (*X*_*i*1_, …, *X*_*iq*_)^′^ for q fixed covariates, including intercepts. For all the methylation levels at *p* CpG sites, we use the methylation beta-values, which roughly represents the fractions of methylated cytosine molecules in the given sample at specific CpG sites. In total, we have data on *n* individuals with $n={\sum}_{i=1}^F{n}_i.$

To derive the test statistic, we first briefly describe how to calculate the score function in a generalized linear mixed model (GLMM), then we describe how to construct the test statistics based on the score vector. A GLMM is an extension of a generalized linear model (GLM) with the addition of 1 or more random effects that are used to account for correlations among the subjects (e.g., members from the same family). Here we use the following model with a random intercept:

$$ g\left(E\left({\mathcal{Y}}_i|{b}_i\right)\right)={X}_i\alpha +{G}_i\beta +{b}_i, $$

$$ b={\left({b}_1^{\prime },{b}_2^{\prime },\dots {b}_F^{\prime}\right)}^{\prime}\sim N\ \left(0,\sum \limits_{k=1}^K{T}_k{\Psi}_k\right), $$

where *g()* is the canonical link function; ${b}_i={\left({b}_{i1},\dots, {b}_{in_i}\right)}^{\prime }$ is the vector of random effects for family *i*; each Ψ_*k*_ is a prespecified *n* × *n* positive-definite correlation matrix (e.g., GRM); and *T*_*k*_ is the corresponding variance component parameter. We estimate the GRM (Ψ_1_) as the empirical correlation matrix based on 20,000 randomly selected single-nucleotide polymorphisms (SNPs). Furthermore, we consider a within-family (shared environmental) effect by adding a block diagonal matrix (Ψ_2_), where each block consists of 1 s for each family. Chen et al. developed R package *GMMAT* to fit the above GLMM, from which we extract the score vector *U* = (*U*_1_, …, *U*_*p*_)^′^ and its corresponding covariance matrix *V* for *p* CpG sites [8].

In GWAS, many existing association tests either directly use the score vector *U* or its asymptotically equivalent counterparts, suggesting that most information is already contained in *U* [6]. As demonstrated in many simulations in the GWAS context [6], depending on the unknown association patterns to be tested, different tests may or may not be powerful. Here, we apply the aSPU test [6] idea and use *U* to construct weights to up-weight the effect of more informative signals. As both SNPs in GWAS and methylation levels in EWAS are treated as predictors in a GLM or GLMM, the extension of the aSPU test from GWAS to EWAS is straightforward with barely any changes; however, the variation of methylation levels changes a lot across the CpG sites. If we treat all CpG sites equally and put a constant weight on the CpG sites, the signal may be masked by some CpG sites with the larger variance. To put more weights on the CpG sites with the smaller variances, we propose using the inverse standard deviation SPUw [7] tests indexed by a positive integer *γ*:

$$ {T}_{SPUw\left(\gamma \right)}=\sum \limits_{j=1}^p{\left({\omega}_j{U}_j\right)}^{\gamma }, $$

where ${\omega}_j=1/\sqrt{\mathit{\operatorname{var}}\left({G}_{ij}\right)}$ is the weight for CpG site *j*, and the SPUw test reduces to the usual sum of powered score (SPU) test if *ω*_*J*_ = 1 for all *j*. Various values of *γ* lead to different SPUw tests that are more powerful under different situations. Hence, for a given scenario with unknown association patterns, we use various SPUw tests to increase the chance of having at least one of them to be powerful. The SPUw tests cover the (weighted) Sum test and sum of squared score (SSU) test as 2 special cases with the corresponding γ = 1 and γ ***=*** 2, respectively. As γ → ∞, the SPUw test would converge to the minimum *p* value (UminP) test [6]. Even though aSPU and aSPUw are originally proposed to analyze GWAS data, as shown in the above formula, once we get the score vectors U and its covariance matrix V from the GLMM, we can apply both aSPU and aSPUw tests directly.

Depending on the underlying unknown association pattern, one of the SPUw(γ) tests may be more powerful. For a given data set, we may gain power if we can select the value of γ for the SPUw tests adaptively. As in Pan et al. [6], an adaptive SPUw test simply combines the results of multiple SPUw tests by taking the minimum *p* values for some candidate values of γ in Γ; for example, Γ = {1, 2, 3,..., 6, ∞}:

$$ {T}_{aSPUw\left(\gamma \right)}=\underset{\gamma \in \varGamma }{\min }{P}_{SPUw\left(\gamma \right).} $$

For the choice of Γ set, we recommend including both small γ values such as 1, 2, and medium γ values such as 3,..., 6 in Γ to maintain high power when the signals are either dense or relatively sparse; in addition, we also recommend including ∞ in Γ to cover the case when the signals are highly sparse.

Note that *T*_*aSPUw*_ is no longer a genuine *p* value, however, we can use a single layer of Monte Carlo simulations [6] to calculate the *p* values for SPUw(γ) and aSPUw simultaneously. Specially, first, we simulate *B* copies of the null score vectors independently, *U*^(b)^ ~ *N*(0, *V*) for b = 1,…, *B*, then calculate the null statistics *T*_*SPUw*(*γ*)_ based on each null score vector *U*^(b)^ accordingly. Next, the *p* value of SPUw(γ) is ${P}_{SPUw\left(\gamma \right)}=\left[1+{\sum}_{b=1}^BI\left(\left| SPUw{\left(\gamma \right)}^{(b)}\right|\ge \left| SPUw\left(\gamma \right)\right|\right)\right]/\left(B+1\right)$, and the *p* value for aSPUw is ${P}_{aSPUw}=\left[1+{\sum}_{b=1}^BI\left(\ {T}_{aSPUw}^{(b)}\le {T}_{aSPUw}\right)\right]/\left(B+1\right)$, with ${T}_{aSPUw}^{(b)}={\mathit{\min}}_{\gamma \epsilon \Gamma}{p}_{\gamma}^{(b)}$and ${p}_{\gamma}^{(b1)}$ =$\left[{\sum}_{b\ne {b}_1}I\left.\left(\left|{T}_{SPUw\left(\gamma \right)}^{(b)}\right|\ge \left|{T}_{SPUw\left(\gamma \right)}^{b_1}\right|\right)\right]/B\right.$.

## Results

We analyzed the GOLDN study data provided by GAW20. The GOLDN study was designed to identify the genetic impact on the lipid response to interventions [3]. Here we tested the association between the log pretreatment fasting TGs and the methylation levels of the CpG sites within each gene’s coding region (without any upstream or downstream extension); we used the identity function as the link function in GLMM (leading to a linear mixed model [LMM]). Furthermore, we adjusted for age, sex, and study center to eliminate potential confounding effects. We included *K* = 2 variance components: first, instead of using the kinship matrix, we used a random set of 20,000 SNPs from the GOLDN GWAS data to estimate the GRM; second, we created a within-family correlation matrix with elements all 1 s for all the members in the same family, and 0 otherwise. We tested 15,731 genes with a conservative Bonferroni adjustment with a genome-wide significance level at 0.05/15731 ≅ 3 × 10^− 6^.

**Fig. 1:**
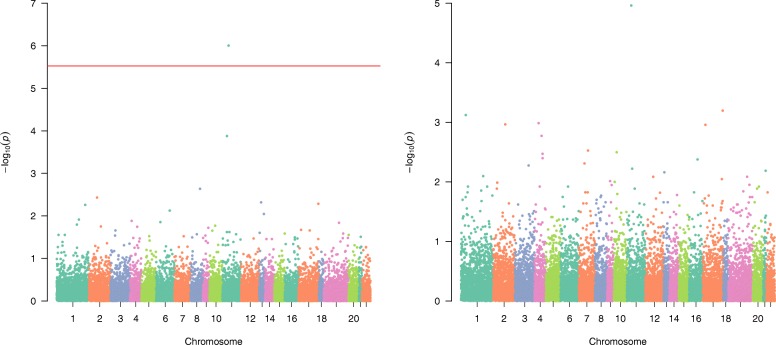
Manhattan plots of aSPUw with the inverse standard deviation weight $\left({\omega}_j=1/\sqrt{\mathit{\operatorname{var}}\left({G}_{ij}\right)}\right)$ *(left)* and aSPU with a constant weight (*ω*_*j*_ = 1) *(right)* applied to the GOLDN study data

Figure 1 shows the Manhattan for aSPUw with inverse standard deviations weight ${\omega}_j=1/\sqrt{\mathit{\operatorname{var}}\left({G}_{ij}\right)}$, and aSPUw (ie, aSPU) with a constant weight *ω*_*j*_ = 1.

Perhaps because of treating all CpG sites equally, aSPUw (ie, aSPU) with a constant weight does not find any significant genes after the Bonferroni correction. In contrast, aSPUw with inverse standard deviations weight identifies one significant gene, *CPT1A*, which is also identified by Irvin et al. [3].

The quantile–quantile (Q-Q) plots (not shown) indicate that all tests yield slightly conservative results (genomic control lambda < 1). Table 1 shows the individual *p* values for 2 genes, *CPT1A* and *APOA5*, with different weights. For fasting TGs, *APOA5* is known to be associated with several significant SNPs, such as rs964184 (*p* value = 7 × 10^− 240^ [9]). Although aSPU, aSPUw, and other individual SPU or SPUw tests are unable to detect *APOA5* at the genome-wide significance level, the *p* values are usually small (*<* 0*.*05). Perhaps as a consequence of the highly sparse signals in *APOA5*, the SPUw or the SPU with a larger *γ* gives a more significant *p* value. The aSPUw test can detect *CPT1A* at the genome-wide significance level, whereas the aSPU (with a constant weight) fails to do so. However, for *APOA5*, aSPU with a constant weight yields a smaller *p* value than aSPUw with the inverse of standard deviation weight. Note that the SPU(2) test is equivalent to the sequence kernel association test (SKAT) with a linear kernel [10]; SPU(2) fails to identify *CPT1A* and *APOA5* at the genome-wide significance level.

**Table 1:** The *p* values of the SPUw and aSPUw tests with different sets of weights

*ω*_*j*_	Gene	SPUw(1)	SPUw(2)	SPUw(3)	SPUw(4)	SPUw(∞)	aSPUw
$1/\sqrt{\mathit{\operatorname{var}}\left({G}_{ij}\right)}$	*CPT1A*	6.2E-01	2.1E-02	4.6E-04	9.0E-06	0.0E + 00	1.0E-06
$1/\sqrt{\mathit{\operatorname{var}}\left({G}_{ij}\right)}$	*APOA5*	9.1E-01	2.5E-02	1.0E-02	1.1E-03	5.3E-05	1.3E-04
		SPU(1)	SPU(2)	SPU(3)	SPU(4)	SPU(∞)	aSPU
1	*CPT1A*	2.3E-01	2.0E-02	1.7E-02	1.7E-02	2.1E-02	3.2E-02
1	*APOA5*	1.0E-02	5.0E-06	5.0E-06	5.0E-06	5.0E-06	1.1E-05

## Discussion and conclusions

In this paper, we applied an adaptive test, aSPU [6], and its weighted version, aSPUw [7], to the GOLDN study data and found 1 significant gene, *CPT1A*, with its methylation levels associated with the log pretreatment fasting TGs. Under different scenarios with differing signal sparsity levels, different tests may be more powerful. Using adaptive testing may achieve overall good performance as we do not know the underlying truth. To alleviate the effects of varying variability of the methylation levels across the CpG sites when conducting a gene-based test, we proposed using the aSPUw test, putting more weights on the CpG sites with smaller variances. Compared to the unweighted aSPU test or other SPU tests (including SKAT), the aSPUw test would be more powerful if CpG sites with smaller variances are more likely to be truly associated with the trait, but may lose power otherwise. More generally, following the idea of the aSPU test in combining multiple SPU tests, it is straightforward and potentially productive to combine various weighted and unweighted aSPUw and aSPU tests, which may maintain higher power across different situations.

There are several limitations in the current study. First, normalization is needed but remains challenging for methylation data. Among others, normalization helps reduce the impact of batch effects, leading to more reliable subsequent analyses. In our current study, we used the given methylation data without any further normalization. Second, because of the page limit, we did not conduct simulation studies to evaluate the performance of aSPU and aSPUw with methylation data, although their promising performance has been shown with extensive simulations and real data analyses in the context of GWAS [6].
